# “Collaboration Toward One Collective Goal”: A Mixed-Methods Study of Short-Term Learning Outcomes and Long-Term Impacts Among Students Participating in an Undergraduate Community-Based Participatory Research (CBPR) Course

**DOI:** 10.3389/fpubh.2021.694840

**Published:** 2021-06-21

**Authors:** Seblewongel Yigletu, Karen C. Kosinski, Alison Kuah, Kenia Alfaro, Ashley C. Holmes, Shalini A. Tendulkar

**Affiliations:** ^1^Department of Community Health, Tufts University, Medford, MA, United States; ^2^Practical Anthropology, University of Cape Town, Rondebosch, South Africa; ^3^Welcome Project, Somerville, MA, United States

**Keywords:** community-based participatory research, CBPR, undergraduate education, students, learning

## Abstract

**Background:** Research shows positive learning outcomes for students participating in service learning. However, the impacts of undergraduate student participation in Community-Based Participatory Research (CBPR) courses are minimally studied.

**Methods:** We used a triangulation mixed-methods design approach to analyze short- and long-term (1–5 years post-course) data collected from 59 undergraduate students across 5 cohorts of a CBPR course (2014–19). Thematic analysis was used to analyze the qualitative data and descriptive statistics and frequencies were generated to analyze the quantitative data.

**Results:** We developed five key themes based on short-term qualitative data: integration of CBPR and traditional research skills; importance of community engagement in research; identity; accountability; and collaboration. Themes from qualitative course evaluations aligned with these findings. Long-term qualitative data revealed that former students gained research knowledge, research skills, and professional skills and then applied these in other settings. This aligns with quantitative findings, where >79% of respondents reported that course participation “extensively” improved their research skills. Post-course, students still reflected on the importance of community engagement in research and reported a substantially enhanced likelihood of civic engagement.

**Discussion/Conclusions:** Students gained critical knowledge and skills that positively impact their ability to engage in community-based work well after the end of course participation. Some students reported considering research-oriented careers and graduate programs for the first time after course participation. Collaborative learning experiences with community partners and members encouraged students to reflect on research designs that center community voices. We stress here that community partnerships require extensive cultivation, but they can create opportunities to translate findings directly back to communities and provide numerous benefits to undergraduate students. We hope that our findings provide the information needed to consider pilot testing practice-based CBPR courses in a variety of public health training contexts.

## Introduction

Service learning (SL), defined as “a structured learning experience that combines community service with preparation and reflection,” has gained increasing prominence in undergraduate public health education as a way to connect academia with communities (1). Many courses engage undergraduates in service learning (1, 2) and studies have focused on undergraduate student outcomes for SL-style courses and initiatives across various disciplines, including public health (3–5). The potential benefits of student engagement in SL classroom settings are numerous. Studies have shown that SL can positively impact students' personal outcomes (e.g., personal identity), social outcomes (e.g., reducing stereotyped thinking), learning outcomes (e.g., ability to apply concepts in a practical setting), career development, and relationships with their institution (e.g., satisfaction) (6). For example, one study showed improvements in student attitudes toward individuals living in poverty (7).

Community-based participatory research (CBPR), a type of SL centered around research partnerships, often begins with a research topic of interest to community partners and involves the community in decision-making around the research process (8). Community-based participatory research is valuable because it allows for the many concerns faced by communities to be better understood and addressed through research (9). For faculty members, particularly those at research-intensive institutions, developing CBPR opportunities for students can enable faculty to maintain research agendas and simultaneously support student learning (10); this can result in numerous benefits, including connecting faculty with researchers and individuals outside of academia and providing new funding opportunities (11).

There are examples of CBPR learning opportunities for graduate professional students (12), and some models from training opportunities for undergraduate nursing students (13, 14). However, we found few examples of undergraduate courses that offer both training in CBPR research fundamentals *and* applications of these lessons through direct and intensive engagement in research by partnering with community organizations and communities (15–17). We found no studies that focused on undergraduate student outcomes and experiences resulting from participation in intensive CBPR courses.

Researchers have called for increased research to understand the impact of CBPR courses given evidence that suggests that they enhance undergraduate students' experiences related to research and help propel them into future research careers (18). We are not aware of any follow-up studies to assess student reflections and practical application of learning outcomes after SL courses have ended. According to the Association of Schools and Programs of Public Health, there are four domains for undergraduate training in public health to support students to “become more active participants in their own and their community's health” (19). While these learning outcomes are critical for undergraduate public health education, Domains #2 “Intellectual and Practical Skills” and #3 “Personal and Social Responsibility” especially relate to CBPR. Domain #2 states that undergraduates should have the capacity to use “collaborative and interdisciplinary approaches and teamwork” to address population-level health challenges. A key CBPR principle is the facilitation of collaborative and equitable partnerships in all phases of the research process (20). Similarly, a key learning outcome in Domain #3 is that undergraduates should learn to “…collaborate with others from diverse backgrounds in addressing health disparities and inequities,” which is strongly emphasized in the practice of CBPR (19). Given the potential value of CBPR in training undergraduates in public health, we assessed students' short-term learning outcomes and long-term impacts (up to 5 years after completing the course) following an undergraduate CBPR course. Our research questions were:

1. What short-term learning outcomes (knowledge and skills) are demonstrated in written student reflection assignments and regular course evaluations completed as part of an undergraduate CBPR course?
2. For former students, what was the long-term (1–5 years later) impact of participating in an undergraduate CBPR course?

## Materials and Methods

### Study Location and Population

Our study focused on students enrolled in a small, private liberal arts college on the East Coast of the United States. The surrounding city had a population of 57,765 residents at the time of the study. People in the community identify as 73.1% white (not Hispanic or Latinx), 8.7% Black, 9.7% Asian, 5.3% Hispanic or Latinx, 0.1% American Indian and/or Alaskan Native, and 3.2% multi-racial, with a 21.6% immigrant population (U.S. Census Bureau, 2018). Median household income is $86,204. Each year, an undergraduate-level (freshmen-seniors) CBPR course is taught at this university by one of the authors as described elsewhere (21). The course focuses on a research project identified by the community partner. The course is designed to provide practical research experience and specifically to teach CBPR.

### Study Design

We used a triangulation research design to answer our research questions (22). We analyzed data from students in all cohorts of the class (*N* = 59) from 2014 to 2019. All participants were approximately 18–22 years old when they were enrolled in the course and mostly female.

### Datasets

We analyzed 7 datasets (Table 1). All the datasets, except for Dataset #4, were routinely collected for assessment and course evaluation purposes. Each dataset is described in additional detail below.

**Table 1:** Description of 7 data sets used for the mixed-methods analysis.

**Dataset**	**Assessment objective**	**Data type**	***N***	**Cohort**
#1a: Student Reflection Assignment	Short-term learning	Qualitative	7	2018
#1b: First Student Reflection Assignment	Short-term learning	Qualitative	15	2019
#1c: Mid-point Student Reflection Assignment	Short-term learning	Qualitative	15	2019
#1d: End-point Student Reflection	Short-term learning	Qualitative	16	2019
#2: Midpoint Survey	Short-term learning	Qualitative	15	2019
#3: Course Evaluations	Short-term learning	Mixed	49	All
#4: Follow-up Survey	Long-term learning	Mixed	34	All

### Short-Term Learning Outcomes

We assessed short-term learning outcomes using datasets #1a through #3.

#### Dataset #1a: First Student Reflection Assignment of the Course

Students in the 2019 cohort (*n* = 15/59 students) completed a first reflection assignment (~2–3 pages long) and were asked to comment on experiences with a classroom discussion activity on identity, privilege, and cultural humility.

#### Dataset #1b: Mid-point Student Reflection Assignment of the Course

Students in the 2019 cohort (*n* = 15/59 students) completed a mid-point reflection assignment (~2–3 pages long) and were asked to comment on experiences with data collection.

#### Dataset #1c: Midpoint Surveys

Students in the 2019 cohort (*n* = 15/59 students) completed a midpoint survey and were asked to comment on (a) how the course is different from other courses, (b) the ways in which they contributed to the research, (c) their most significant accomplishment, (d) content- or application-related areas where they struggled, (e) general feedback, and (f) the grade they would give themselves at that point in the semester.

#### Dataset #1d: End-Point Student Reflection Assignment of the Course

Students in the 2019 cohort (*n* = 16/59 students) completed an end-point reflection assignment (~3–4 pages long) and were asked to connect the CBPR work during the semester to CBPR principles and discuss the extent to which the research team engaged authentically in CBPR.

#### Dataset #2: Student Reflection Assignment

Students in the 2018 cohort (*n* = 13/59) submitted one “Interview/Reflection” assignment with a choice to either reflect on their participation in the course or complete an interview with an academic or community partner who conducts CBPR. We used only data from reflection assignments (2–5 pages long) in this analysis (*n* = 7/13) because the interview write-ups did not include explicit student reflections on their own experiences. One student did not complete either assignment.

#### Dataset #3: Student Evaluation

Dataset #3 consisted of regularly collected, standardized, anonymous, university course evaluations (2014–19). Evaluations were completed by 71–92% of students in each cohort. The evaluations asked for student feedback on the course was taught, and the strategies used by the instructor. Course evaluations are anonymous, so they were matched with other data sets at class level rather than student level.

### Long-Term Learning Outcomes

#### Dataset #4: Follow-Up Survey

We used dataset #4, collected via Qualtrics, to assess long-term retention of key CBPR learning outcomes. We also assessed whether students reported that participation in the course impacted their professional growth, personal growth, and present-day civic engagement. Lichtenstein et al. (23) developed a tool called the Student Learning Outcomes Survey of Community-Based Research to measure student learning outcomes in Community-Based Research (CBR) courses. We adapted this survey slightly to include questions about the participatory aspects of CBPR. Our survey included 25 close-ended Likert-scale questions in six domains: (1) academic skills, (2) educational experience, (3) civic engagement, (4) professional skills, (5) personal growth, and (6) knowledge of a local community. It also contained four open-ended questions about academic, professional, and civic engagement. We contacted all former students of the CBPR course (*n* = 59) up to three times and gave all participants 2 weeks after the initial email to complete the online survey. The survey was not anonymous so data could be matched with data from earlier timepoints. Following matching with earlier data, the surveys were deidentified and assigned an ID number prior to data analysis.

### Quantitative Data Analysis

We summarized student and class information and calculated frequencies for course evaluation data. We examined long-term learning outcomes across the long-term survey domains and calculated frequencies for the items within each of the six subscales in the long-term survey. We also calculated alpha coefficients for each of the six subscales in the long-term survey, which ranged from 0.70 to 0.91. We analyzed data using Stata (Version 16.1) (24).

### Qualitative Data Analysis

We thematically analyzed (25) qualitative data in all seven datasets. All coding was done in Nvivo (26). To code student-level qualitative data, we deductively approached codebook development. This included creating a codebook based on key course concepts and anticipated student learning outcomes. Next, we coded two randomly selected transcripts that contained all available qualitative data for each student. The authors convened and reevaluated the codebook, which did not fully capture the breadth of learning outcomes evident through student assignments. Next, we conducted a reflexive reading of the two randomly selected transcripts and independently developed revised lists of codes. Then we met, discussed, and combined codes into one codebook, which included a list of 10 inductive parent codes and 19 child codes. We then used the revised codebook to code the transcripts for three additional randomly selected participants and discussed coding discrepancies and made minor modifications to clarify definitions and collapse codes. Finally, we independently coded all additional transcripts and discrepancies in coding were verbally reconciled. When new codes were added to the codebook, all the transcripts were re-reviewed.

After independent data coding, we merged the codes into one set of transcripts and met to discuss discrepancies in coding and reconcile the codes to generate one final transcript for each participant. For a small portion of the qualitative data, one author coded the data independently at this stage. We then reviewed all coding reports to check for errors.

We reflected on codes individually and created initial themes based on similarities. We then collaborated to find connections between themes and to develop our global, overarching themes.

### Protection of Human Subjects

This study was approved by the Tufts University Institutional Review Board as expedited on July 17th, 2019. All former students of the course were informed that participation in the study was optional and had no bearing on future educational endeavors. We assigned random ID numbers before data analysis and only analyzed deidentified data. For participants who reflected on their long-term learning outcomes, we collected names but decoupled names before data analysis. We also provided a separate form that was anonymous for any additional feedback. We used student data only if we received affirmative consent.

## Results

### Student- and Class-Level Demographics

Overall, 59 undergraduate students participated in this course from 2014 to 2019 (Table 2). Most (>70%) participants were juniors or seniors and most (>66%) participated in a half-year version of this course.

**Table 2:** CBPR course student- and class-level demographic information for cohorts from 2014 to 2019.

****	***N***
**Enrollment by class cohort**	59
2014-2015 (full-year course)	7
2015-2016 (full-year course)	13
2017 (half-year course)	10
2018 (half-year course)	13
2019 (half-year course)	16
**Overall class year composition**	
Freshman	4
Sophomore	13
Junior	22
Senior	20
**Course length**	
# of students in half-year course	39
# of students in full-year course	20
**Research project type**	
# of students who completed a Qualitative project	52
# of students who completed a Mixed Methods project	7

### Key Themes Based on Short-Term Qualitative Data

We developed five key themes based on short-term qualitative data: Integration of CBPR and Traditional Research Skills (Theme S1), Importance of Community Engagement in Research (Theme S2), Identity (Theme S3), Accountability (Theme S4), and Collaboration (Theme S5).

#### Theme S1: Integration of CBPR and Traditional Research Skills

Students have many opportunities to acquire traditional research skills in the department and university where this course is based; however, this course teaches students to build on the traditional research framework by incorporating CBPR principles and values. In the written reflections that students completed during the course, students described all the major aspects of the traditional research framework with an emphasis on three components: data collection, data analysis, and research findings. Students showed the ability to simultaneously discover and incorporate CBPR principles and values into the traditional research process. This integration was evident in student comments about developing research knowledge and skills with a specific focus on considering the community and community needs.

##### Sub-Theme S1.1: Data Collection Skills

Many students noted participating in and gaining practical experience with data collection. Notably, students expressed appreciation for the utility of qualitative data as a vehicle for revealing community members' stories, as noted by Participant #5,

“I think this process and this class has opened my eyes to the importance of qualitative research, especially in communities whose voices aren't typically heard from. Narratives and stories are how our brains are wired, and qual research is an amazing way to understand experiences better.”

Furthermore, multiple students described how interview data quality and quantity were related to factors typically discussed in the literature such as types of questions asked, interviewers' tone of voice, interviewers' eye contact, and listening strategies, but they also pointed out additional factors such as language barriers, and the positionality of the interviewer in relation to the community participant. These factors were heavily emphasized by the course instructor, given the CBPR focus.

##### Sub-Theme S1.2: Data Analysis Skills

Nearly all students wrote about data analysis, and several aspects were noted as being time-intensive and challenging. Students who transcribed qualitative data described the time-consuming nature of transcription. Other students focused on the difficulty of coding qualitative data rapidly and described a learning curve for the coding process not just for students but also community partners who were engaged in the data analysis process. For example, Participant #2 wrote:

“I think one area where I have struggled is to understand the coding process fully, because this is a process that I have never been involved with before. This is definitely an area of growth for me because it is something I would love to further understand and be able to do sufficiently, and this is a perfect environment to enable this learning.”.

Several students commented on the need for a lengthier timeline for data analysis. In general, students in the 1-semester version of the course consistently indicated the need for increased course length.

##### Sub-Theme S1.3: Research Findings

Students reflected on their research findings in different ways, ranging from recall of and reflections on specific participant comments to descriptions of overarching findings. When students reflected on specific research findings, they often discussed comments from interview participants or compared information shared by two or more participants. When students described findings from the aggregated data, they often tied those findings to the need for actionable next steps (e.g., the capacity of the community partner to apply the findings) and translational work, which is emphasized in CBPR and would result in tangible impact and benefit for the community. We describe this aspect of the theme further below. Finally, some students recognized the limitations of their findings and discussed how the validity of the findings depended on things like sample size, sample composition, and the sophistication and capabilities of their data analysis.

#### Theme S2: Importance of Community Engagement in Research

Many students described growth in their understanding of CBPR and a newfound appreciation for the importance of community engagement in research. Based on their exposure to both the theoretical and practical aspects of CBPR, students articulated four ways that community participation in research improves the quality and impact of research: (1) community stakeholders have key information and they may be the best or only source of this information; (2) communities have better access to local resources than outsiders; (3) community participation provides opportunities for student and community co-learning; and (4) findings can be more impactful because community engagement facilitates knowledge translation.

Participant #3 touched on the theme of co-learning, noting:

“Not only did we learn a lot about [the community] and its educational system, but our collaborators such as the [Partner Organization] and the youth from the [Partner Organization Interpreter Program] learned a lot about conducting research through our collaboration.”

Similarly, Participant #4 commented on the unique resources and knowledge of the partner organization:

“While we did the work, [Partner] helped give us some direction. For instance, initially when drafting our interview guide there was talk about asking a lot of demographic questions or a pre/post interview survey where we collected demographic information. However, [Partner] helped us realize that categorizing these people right away instead of just listening to them may not make for the best interview, nor is it really the information that community is looking for…”.

#### Theme S3: Identity

Students reflected on experiences in the course that encouraged them to think more deeply about their identity in relation to other students, community members, and partners. For example, Participant #1 signed up to co-facilitate the classroom session on identity, recognizing the importance of this topic:

“I was looking forward to this session because I think that understanding one's own identity and how it relates to those around you is crucial to understanding one's self and role in life. This is especially relevant when doing research in communities of color with a group of students that are not from the community, many of whom are not people of color. I think that the work we are doing is can be very valuable but I do wi[s]h that there were students from [Host City] in our class.”

Most students acknowledged that reflections on identity were important because it prepared them for CBPR and participant engagement. Participant #6 wrote, “…the participants we interview are likely going to be opening themselves up and exposing aspects of their identities to us, I think it is only right that we the researchers make an effort to come to terms with our own identities.”

Other students commented on how their identities conferred privileges, with Participant #4 noting,

“When we are going out into the community and working with vulnerable populations it is extremely important to reflect on the things that we may take for granted. Everyone in the class at least experiences the privilege of a college education. And not only a college education, but a college education at a highly ranked private institution. Additionally, many students in the class experience other privileges as well.”

Some students recognized that shared identities, namely language and to a lesser extent cultural background, either enabled them to connect with participants or created distance between them and participants. Participant #11 noted the difficulty of building rapport with participants across a language barrier, writing, “Having taken qualitative research, I have some experience with consent forms and interviewing.but the added element of a language barrier certainly made the establishment of rapport more difficult.”

While, some students worked with community interpreters to overcome language barriers during data collection, some students felt that working with an interpreter made the process of interviewing more challenging. Participant #4 shared, “I also felt like there was a little less flow when I was using an interpreter. Due to the fact that the responses had to be translated back to me I felt like I couldn't jump in with questions as easily as I could when I did an interview in English.” Others noted that working with an interpreter facilitated data collection. Participant #1 wrote, “The first person I interviewed was a Brazilian mother…It was helpful to have an interpreter because it allowed more time for me to write down notes without having long pauses with silence.” Participant #5 commented on the complexity of relationship-building even with shared language capacity, noting:

“The interview I conducted in [Language] was also an interesting learning experience because the parent and I aren't from the same country, and that the connection I had built with her was mostly through sharing the same language as opposed to maybe also cultural aspects that others native interviewers may have had.”

Finally, Participant #14 emphasized the importance of reflection on commonalities and differences in identity:

“Although we may not share many similarities in identity, I think I will be able to appreciate the differences and also understand that we may share similarities in our thoughts. I also feel like I do not necessarily have to hide the privilege background that I come from but that I need to get in the mindset to understand their background. I am really excited to hear their stories.”

#### Theme S4: Accountability

In the short-term, many students across all cohorts discussed various forms of being accountable to community partners and community members (Table 3). Accountability was discussed in three specific ways: (1) preparing oneself to partner with and learn from community partners, which we call “personal preparation”; (2) working to ensure the access, use, and translation of data by community members, which we refer to as “non-extractive partnerships and translational sciences”; and (3) ensuring partnerships were maintained beyond the end of the course, which we denote “sustainable partnerships.”

**Table 3:** Sub-themes and illustrative quotes within the larger theme of accountability.

	**Participant #**
**Sub-theme #S4.1 Personal Preparation**	
One aspect of this class.that initially scared me a lot…[were] my worries of being patronizing, of our class not being respectful or equipped enough to understand the participants, and not feeling qualified or worthy of being on this research team, for both personal identity and education reasons.	2
**Sub-theme #S4.2 Non-extractive Partnerships and Translation**	
This project was very different though and felt much more valuable because we were not only adding to the knowledge base, but also performing a direct service to the community and attempting to fill a need that community members cite as being a problem.	25
…[Our] team really stressed disseminating what we learned, as well as recommendations, to the participants at a time when they'd be available in the evening…In addition, the team really stressed giving the participants something concrete to take back with them.	30
**Sub-theme #S4.3 Sustainability**	
One of the other core components of our research project was what was to come after. An additional tenet of CBPR is to ensure planning is done for the long-term, and that anything implemented is sustainable. I believe we exceeded expectations in this area, not only providing data to be used for one intervention, but as a basis for future research and several programs or interventions which were already under consideration.	16
…In our participation in this research partnership, have we worked to build a strong enough framework for action to improve immigrant life in [Host City]? What more could/should we have done to ensure that action is taken following our research? .I am curious about how collaboration will continue…and how the lack of continuity over the summer and in the fall semester will impact the outcomes of the research and the action taken…	18

##### Sub-Theme S4.1: Personal Preparation

This sub-theme reflects student ideas about accountability to community partners by preparing oneself adequately for data collection. Students discussed preparation in two ways: acquiring and refining traditional research skills to complete the research professionally and reflecting on personal factors that might affect engagement with community members. Student comments about improving traditional research skills mainly focused on data collection, and specifically how to conduct interviews appropriately and how to interact with community members and interpreters. For example, Participant #18 noted:

“…one area of improvement for the whole team, myself included, would be the lack of interpersonal engagement with the focus group participants and the [Community Partner] translators. In the time before the focus group participants began to arrive, we were sitting around the tables, but our translators from the [Community Partner] were sitting in another part of the room and no one from our class spoke with them. I didn't realize this until towards the end of that stretch of time, and really regretted not trying to engage with them more. I felt uncomfortable with this separation between us; it made the environment (at least from my perspective) much less participatory and much less like an equitable partnership. I do wonder what caused this divide—and what could be done to ensure this does not happen in the future.”

Many other students commented on personal preparation to do the work by considering their future behavior: they planned to speak less, listen more, allow silence, make eye contact with participants, take better notes, and carefully record participant comments. Students also recognized the importance of self-reflection as a preparatory strategy, as noted by Participant #6:

“After filling in my information for each bubble [in the identity activity], I definitely felt like I had a more wholesome and well-rounded understanding of my own identity. I wasn't paying enough attention to considerations such as socioeconomic status, the type of geographical setting I was raised in, my education status, my sexual orientation, among other things. When going into a setting where I am dealing with people from backgrounds different from my own, I think it's extremely imperative that we, as the interviewers, have a comprehensive understanding of our own identities to help us think about some potential boundaries that we should be conscious of during the interview process.”

Participant #5 expressed similar sentiments, noting:

“Filling out the [identity] wheel was something that I had done variations of throughout my time at [University Name]. I had been surprised to learn that I had become more comfortable with talking about social identities after so long of being extremely uncomfortable with it. However, though I felt more comfortable with the vocabulary and historical context of my identity and expressing it, I still believe that it doesn't necessarily make me more equipped when I interact with ‘real world people.' I often feel a gap between what I know about myself and how I feel and knowing how to use that knowledge to better my interactions with others. And at the end of the day, vocab and identity reflections like these, are very limited to spaces like [University Name] (as [Community Partner] said) and the important thing is how I outwardly present these attitudes.”

##### Sub-Theme S4.2: Non-Extractive Partnerships and Translational Sciences

Students consistently expressed a desire to create non-extractive relationships; they very clearly called for data to be collected in the community, analyzed with partners, and then used to create tangible impacts for the Host Community. Students understood the importance of the work from an academic standpoint but emphasized accountability to community partners and community members, as noted by Participant #6:

“Yes, this research can be valuable to the larger academic community who may come across our findings on some database, but the purpose and motivation behind this research was to help address the inequities in [Host Community] specifically, and informing the parents and other community members about our conclusions [is] crucial in accomplishing that.”

Students talked about the wide variety of ways that they disseminated their findings, as noted by Participant #18:

“Lastly, we disseminated results to all partners and involved them in the wider dissemination of results. We created a PowerPoint, poster, and a 2 pager, and we presented our findings to [Community Partner]. In addition, we worked closely with staff members from the community in order to frequently update them on our progress. I believe following this principle allowed us to cater towards the partner's needs better and consequently alter our research process.”

Some students were concerned about insufficient time for dissemination activities during the one-semester version of the course and whether this would affect the ability of community partners to use the findings, as noted by Participant #25:

“I worry how much impact this project will be able to have because we just don't have the time necessary to extract all of the insights out of our interviews and focus group, and put it together in a meaningful way to make precise recommendations for the community and [Community Partner]. Something that I felt discouraged by in this project was that a lot of the feedback we obtained from the focus group, although important, was not necessarily actionable. It is difficult for me to hear all about the various struggles these immigrant families have and to not have a good solution or way to make li[f]e better for them in any significant way. It makes me unsure of what our next steps should be in terms of having a meaningful contribution to report back to the community.”

In general, many students talked about knowledge translation and accountability to the Host Community, acknowledging these as distinguishing features of the course and commenting on their importance. Participant #1 noted, “The main difference from other courses is that we are actually doing work that can benefit the community, instead of just learning in ways that only benefit ourselves (at least in the short term).”

##### Sub-Theme S4.3: Sustainability

Finally, students focused on long-term accountability to the community by the university and its students. Students described the importance of continuing the work after the end of the course and focusing on overall sustainability. Participant #16 commented on this:

“An additional tenet of CBPR is to ensure planning is done for the long-term, and that anything implemented is sustainable. I believe we exceeded expectations in this area, not only providing data to be used for one intervention, but as a basis for future research and several programs or interventions which were already under consideration. I felt as though at least a great portion of the class had the intent to build a foundation on which others could continue to develop further, more targeted, approaches for issues which were brought up.”

#### Theme S5: Student-to-Student and Student-to-Community Partner Collaboration

Most students recognized that collaboration with other students and community partners was a key distinguishing feature of this course and meant a shift from thinking about the needs of individuals to considering the needs and goals of the group. The first way that “collaboration” was a point of emphasis in the transcripts was through the contrast of “Individualism” vs. “Collectivism.” Second, students mentioned countless ways in which the course helped them develop concrete collaboration skills, which we refer to as “collaboration skill-building.” The benefits of community collaboration, specifically, were described earlier in Theme 1, “Importance of Community Engagement in Research.”

##### Sub-Theme S5.1: Individualism vs. Collectivism

This course's emphasis on teamwork and collaboration with the student team and with community partners was described by many students as a distinguishing feature. As noted by Participant #14, the course “…is extremely collaborative where we have to work closely with our teammates and teachers…and it isn't confined to the classroom—it extends within our community.” Many students articulated that it was important for them to think about group or collective goals rather than focus only on themselves. As articulated by Participant #5, “It is a course in which student learning is not the main goal; we…learn to work with a community partner to conduct research in a community.” Participant #6 commented on collective goals: “The main aspect of the course that differs from any class I've ever taken is that we work in a small, more intimate environment…I have never been in a course where we worked in collaboration towards one collective goal.”. A collectivism mentality is specifically fostered in this course by the instructors, rather than encouraging an individualistic mindset.

##### Sub-Theme S5.2: Building Collaboration Skills

Several students discussed developing teamwork and collaboration skills through engagement with team members and community partners. Participant #6 shared, “I think I have really developed my skills involving group collaboration and what that encompasses (communication skills, attendance/punctuality, organization, planning, etc.).” Students also described practicing how to respect other viewpoints and balance the differing perspectives and backgrounds of their partners and teammates while still making progress toward a shared goal. Some of these themes are illustrated by Participant #25:

“Additionally, though I know it is crucial to treat all of our community partners as part of our team, I couldn't help but feel that we were losing efficiency by having to coordinate and check everything through multiple organizations and not simply making decisions on our own as to how to proceed with the project. Certainly, it has been frustrating trying to manage multiple schedules, priorities, and deadlines, but it was clear that everyone brought something different and valuable to the project. It was difficult for me to not be in control of all aspects of the project, but I had to remind myself that it would ultimately create a better outcome. This somewhat deferral of responsibility and decision-making was quite the learning experience for me, and it helped me to find a balance between when to step up and when to step back.”

During the course, only two students described applying teamwork and collaboration skills in other settings, but after completing the course, students thought broadly about how and where they apply these skills (See below).

#### Course Length

Beyond these five key themes, many students commented on the length of the course and described that time constraints challenged every aspect of the research process, and in particular, time constraints caused struggles with data analysis and the dissemination of findings. Student writing did not show a recognition that time constraints are common in research in general; instead, they viewed the time constraints as more particular to this specific partnered research endeavor and this course.

### Key Themes Based on Long-Term Qualitative Data

We developed three key themes related to the long-term impact of participating in the course: (a) application of traditional research skills and knowledge to other contexts; (b) application of professional skills to other contexts; and (c) understanding of the importance of community engagement in research.

#### Theme L1: Application of Traditional Research Skills and Knowledge in Other Contexts

After completing the course, many students reported continuing to use traditional and CBPR research skills in contexts such as academic settings, internships, and jobs. Former students discussed mainly applying data collection, data analysis, and writing skills, and to a lesser extent, partnership and IRB skills. In general, there was a strong, consistent message of feeling well-prepared for post-course data collection and analysis experiences, as Participant #50 explained:

“I am currently a senior research analyst at a health market research firm and the analytical skills I learned…are applied every single day. Majority of my focus is on the analysis; we conduct quantitative and qualitative research using various methodologies (ethnographies, online surveys, telephone interviews) and this course really prepared me for not only being exposed to that kind of research, but the analytical thinking/processes that goes with it.”

Ten students noted that writing skills they developed in the course enabled them to more effectively contribute to written products (e.g., posters, research proposals, manuscripts) in a professional context, particularly if they have pursued graduate school or research-oriented careers. For example, Participant #25 wrote, “My current job involves a combination of writing up protocol documents and direct communication with our patient users—the written communications skills this class helped me developed have been extremely crucial in success in both aspects of my job.”

#### Theme L2: Application of Professional Skills in Other Contexts

After completing the course, many students reported continuing to use professional skills. We categorized commonly mentioned professional skills as either administrative skills or interpersonal skills. Administrative skills included email etiquette, organization, planning, and budgeting, among others. Interpersonal skills involved (a) communication and listening, (b) advocacy for self and others, (c) respect for multiple viewpoints, (d) and teamwork/collaboration skills. For example, with respect to communication and listening skills, Participant #19 shared:

“I use so many of the professional skills I learned in [Course Name]…[in] my job as a Clinical Research Coordinator. I utilize my teamwork skills. I put into practice the communication and listening skills I developed in [Course Name] as I form relationships with patients and listen to their experiences in the clinic and research community.”

Participant #40 wrote about improving their listening skills when working with teams:

“One of the best professional skills I learned from [Course Number] was the seeking input from all team members before making a decision, even if you do not think that their input [is] necessary. This has allowed me to make more cohesive teams and also create outcomes that I would have never expected.”

Participant #5 discussed how the course fostered self-advocacy skills, writing that the course “…taught me a lot about how to work in a professional environment and to advocate for my views to be listened to.” Students described applying their teamwork and collaboration skills to other settings, including sports teams, student groups, jobs, internships, graduate school, research teams, and private companies. Finally, in reflecting on their experience in this course, several students noted fundamental changes in themselves. Participant #35 noted that the course “…increased my empathy and showed me the importance of being involved in my local community.”

A small percentage of students reported not specifically learning “professional skills” in the context of the course; they may have felt that they already had these skills, or they may not remember them being specifically taught.

#### Theme L3: Understanding of the Importance of Community Engagement in Research

Students continued to reflect on the importance of community engagement; they discussed gaining a conceptual understanding of CBPR, and the benefits of actively practicing CBPR. Students found that this course, in contrast with other undergraduate courses, allowed them to witness ways that academic research findings can directly impact surrounding communities and provide tangible benefits. A response from Participant #18 shows that practicing CBPR principles throughout the course led to an increased appreciation for active collaboration with communities and resulted in improved civic engagement. This participant also reflected on the fact that research could prioritize both the researchers' goals and the community's needs.

“The knowledge I gained of the [University] host community during [Course Number] was incredible and has led me to value it so much more than before I took the class. I've deepened my involvement in student organizations that are civically and community-service oriented. The class also showed me that research does not only have to be exclusively for knowledge-gaining, but also that it can contribute to positive action being taken, action that uplifts communities and prioritizes their needs and wishes. It's made me feel hopeful that if I am to pursue a career in research, that research can be done meaningfully, oriented towards service and action.”

### Quantitative Long-Term Outcomes

A total of 34 students completed at least 12 out of 13 questions on the long-term learning outcomes survey (Table 4). Most (79.4%) respondents reported that participation in the course “extensively” improved their research skills; most (70.6%) reported that course participation “extensively” increased their interactions with faculty, and most (67.7%) reported that the course “extensively” enhanced the likelihood that they will participate in civic activities. Similarly, across the professional skills, personal growth, and knowledge of local community domains, >70% reported that the course “extensively” improved their ability to work on a team, close to 60% reported the course improved their ability to consider other perspectives, and around 75% reported an enhanced understanding of health issues facing local communities.

**Table 4:** Long-term outcomes assessed through dataset #4 (*N* = 34).

	**Extensively**		**Moderately**		**Minimally**		**Not at All**		**Missing**	
**Domain**	***N***	**%**	***N***	**%**	***N***	**%**	***N***	**%**	***N***	**%**
**Academic**										
Improved research skills	27	79.4	7	20.6						
Enhanced understanding of academic learning through other courses	25	73.5	6	17.7	3	8.8				
Strengthened analytical skills	19	55.9	13	38.2	2	5.9				
Improved academic writing skills	4	11.8	24	70.6	6	17.7				
**Educational Experience**										
Increased faculty interactions	24	70.6	9	26.5					1	2.9
Increased major interest	25	73.5	7	20.6	1	3.0			1	2.9
Increased interest in graduate school	14	41.2	11	32.4	5	14.7	3	8.8	1	2.9
Clarified career path	7	20.6	17	50.0	5	14.7	4	11.8	1	2.9
**Civic Engagement**										
Enhanced likelihood I will participate in civic activities	23	67.7	8	23.5	2	5.9			1	2.9
Helped me empathize with those from a different background	20	58.8	11	32.4	2	5.9			1	2.9
Deepened understanding of others not like me	19	55.9	13	38.2	2	5.9				
Helped clarify values	18	52.9	12	35.0	3	9.1			1	2.9
Enhanced likelihood I will vote	10	29.4	13	38.2	9	26.5	1	2.9	1	2.9
**Professional Skills**										
Improved ability to work as part of team	24	70.6	7	20.6	2	5.9			1	2.9
Improved ability to delegate	12	35.3	14	41.2	7	20.6			1	2.9
Improved ability to listen to others	19	55.9	11	32.4	2	5.9	1	2.9	1	2.9
Improved conflict resolution skills	10	29.4	10	29.4	12	35.3	1	3.0	1	2.9
Improved ability to facilitate meetings	19	55.9	10	29.4	4	11.8			1	2.9
**Personal Growth**										
Improved ability to consider others perspectives	20	58.8	12	35.3	1	2.9			1	2.9
Deepened understanding of myself	14	41.2	10	29.4	9	26.5			1	2.9
**Knowledge of Local Community**										
Enhanced understanding of health issues facing host communities	25	73.5	8	23.5	1	2.9				
Enhanced understanding of school's positionality within host communities	22	64.7	9	26.5	3	8.8				
Enhanced understanding of resources within host communities.	21	61.8	12	35.3	1	2.9				
Enhanced understanding of organizational landscape within host communities.	20	58.8	8	23.5	6	17.7				
Enhanced understanding of policy environment within host communities.	13	39.4	11	33.3	7	21.2	2	6.1		

### Quantitative Course Evaluation Data

Quantitative course evaluation data from students in all cohorts showed that > 93% of students rated the course as “excellent” or “very good” across most course and instructor evaluation domains, with the exception of “out of class activities,” which was rated as “excellent” or “very good” by over 85% of participants. None of the students rated the course or the instructor as “satisfactory” or below across all cohorts. We interpreted these findings as evidence that students can recognize the learning opportunities that are presented by facing a variety of practical challenges in a hands-on CBPR course.

## Discussion

Our study is the first to demonstrate both short-term learning outcomes and long-term impacts of a practice-based CBPR course for undergraduate students. Despite variation in the course model over time (e.g., length of course, assignments, etc.) the course instructor consistently used two key pedagogical strategies across all cohorts of students. Specifically, she employed active learning techniques to teach research skills and she used team-based learning where community partners were an extension of the research team and were also actively engaged in providing feedback and training to students in the classroom. Our study shows that students across the five cohorts described similar types of learning and reported similar types of growth and skill development. In the short-term, our data show a positive impact on the personal development of undergraduates, including an ability to understand and collaborate with others, and to recognize of the importance of accountability to community partners and community members, all important transferable skills associated with a liberal education in public health (27). In the long-term, students discussed applying CBPR skills to professional, educational, and extracurricular contexts, and they consistently stated the importance of community engagement in public health.

It is increasingly common for active learning to be incorporated into undergraduate courses; for example, instructors across many STEM disciplines engage students through course-based undergraduate research experiences, and this type of learning has been associated with positive student outcomes such as improved exam scores and graduation rates (28–30). In other disciplines like public health, active learning, in the form of service learning or experiential learning opportunities also demonstrate positive student learning outcomes (5). However, to the best of our knowledge, when active learning is integrated into community and public health courses, it often either involves short-term service-learning opportunities consisting of a few hours of community-based work (5) or is structured to prioritize student learning with less of an emphasis on centering community needs (31). When students do participate in more sustained course-based active learning experiences, this is often through opportunities like individual independent studies, lab-based experiences, or theses that are limited because they are available to limited numbers of students. Furthermore, there are fewer studies of student learning outcomes and impact associated with course-based service learning that takes the form of CBPR (12, 16, 17, 32, 33).

Offering active learning opportunities through course based CBPR partnerships to undergraduate students presents key benefits. First, involving students in research-based active learning can allow students to gain and practice traditional research skills (34) and lead to other academic successes including supporting professional pursuits (35). This may be especially critical for students from underrepresented backgrounds, given extensive discussions about creating a pipeline for research careers (36). Students in this course frequently wrote about learning concrete research skills and they continued to discuss the application of these skills in other contexts after course completion. For example, depending on their cohort, students had opportunities to engage in academic dissemination of their work through an undergraduate research symposium and a professional public health conference. Students who described participating in academic dissemination wrote about enjoying the process because they saw the broader impacts of their work and recognized the opportunity to reach a wider audience. This type of experience helped some students consider research-oriented careers for the first time. Our findings show that students have pursued graduate school and careers in research and CBPR; they report retaining information from this specific course and applying that in their current positions.

A second key benefit to active learning occurs when courses are offered with a team-based component. We have shown here that this collaborative element may promote the development of important professional skills. Some of the key features of team-based learning, namely intentional formation and management of teams and frequent, timely, instructor feedback and accountability (37) were incorporated across all cohorts of this class. Students consistently mentioned collaboration and the development of collaboration skills as important learning opportunities in this course. While there is limited literature on the impact of team-based learning in undergraduate public health settings, studies from other allied-health disciplines, including pharmacy and medicine, demonstrate important outcomes of this type of work, including improved student engagement and the development of communication and critical thinking skills (38, 39).

Finally, team-based active learning experiences that extend into the community through CBPR can encourage students to think about how the research process should be designed to incorporate community partners and community members, and how to center community voices. While incorporating community participation into an undergraduate course requires considerable investment on the part of the instructors and community partners, the learning experience can be beneficial to all. For students, working with community partners on real-world projects can enable them to apply classroom learning to a community-based setting, gain other professional skills like communication and promote a sense of social responsibility and civic engagement (17). Community partners can benefit through access to research resources such as software and scholarly databases, and it can ultimately promote research agendas that address community interests (40). While these types of partnerships require extensive cultivation, they provide opportunities for co-learning between students and communities, and for translating findings directly back to the community.

There are few examples of courses that offer training in research fundamentals that also apply these concepts through direct engagement with surrounding communities (15, 17, 41). This is not unusual given that orchestrating these kinds of learning experiences in an undergraduate context poses some unique challenges. Instructors can find it very challenging to provide a meaningful learning experience within the context of a one-semester course. Notably, many students in our study commented on the challenges of conducting data analysis and disseminating findings within one semester. We hope that our findings provide information to public health educators so that they can consider options for course goals and learning objectives, and they can plan accordingly for partnership development before beginning a CBPR course. We hope that our findings provide enough information for administrators to consider pilot testing practice-based CBPR courses in a variety of public health training contexts.

To ensure the credibility of our data analysis, the two researchers performing most qualitative data analysis brought varying perspectives: one is a former student of the course and the other is an educator unconnected to the course. Additionally, the course instructor participated in codebook generation but not in most qualitative data analysis.

Our study is limited slightly by the fact that we lack access to many demographic variables. While, other studies (23) have shown no differences in learning outcomes by sex, race, or socioeconomic status for (17) community-based research learning experiences, future studies should examine additional demographic variables.

## Data Availability Statement

The raw data supporting the conclusions of this article will be made available by the authors, without undue reservation.

## Ethics Statement

The studies involving human participants were reviewed and approved by Tufts Social, Behavioral & Educational Research IRB Office. The patients/participants provided their written informed consent to participate in this study.

## Author Contributions

SY, KK, and ST contributed to conception and design of the study and wrote sections of the manuscript. ST, KA, and AK co-taught the CBPR course that is described in this manuscript. SY and KK performed the majority of the qualitative data analysis with support from ST. ST performed the quantitative data analysis with support from AH. AH, KA, and AK contributed to manuscript revision, read, and approved the submitted version. All authors contributed to the article and approved the submitted version.

## Conflict of Interest

The authors declare that the research was conducted in the absence of any commercial or financial relationships that could be construed as a potential conflict of interest.

## References

[1] Cashman SB Seifer SD. Service-learning: an integral part of undergraduate public health. Am J Prevent Med. (2008) 35:273–8. 10.1016/j.amepre.2008.06.012 18692742

[2] Anderson LS Royster MO Bailey N Reed K. Integrating service-learning into an MPH curriculum for future public health practitioners: strengthening community-campus partnerships. J Public Health Manage Pract. (2011) 17:324–7. 10.1097/PHH.0b013e3182140bcc 21617407

[3] Daniel KL Mishra C. Student outcomes from participating in an international STEM service-learning course. SAGE Open. (2017) 7:1–11. 10.1177/2158244017697155

[4] Decker K Hensel D Fasone L. Outcomes of a bystander intervention community health service-learning project. Nurse Educ. (2016) 41:147. 10.1097/NNE.0000000000000232 26633150

[5] Mason MR Dunens E. Service-learning as a practical introduction to undergraduate public health: benefits for student outcomes and accreditation. Front Public Health. (2019) 7:63. 10.3389/fpubh.2019.00063 31001507 PMC6454065

[6] Eyler J Giles D Stenson C Gray C. At A Glance: What We Know about The Effects of Service-Learning on College Students, Faculty, Institutions and Communities, 1993- 2000 (No. 139). (2001). Available online at: https://digitalcommons.unomaha.edu/slcehighered/139/

[7] Proctor P Lake D Jewell L Racine L D'Eon M Reeder B. Influencing student beliefs about poverty and health through interprofessional community-based educational experiences. J Res Interprofession Pract Educ. (2010) 1:145–58. 10.22230/jripe.2010v1n2a24

[8] Minkler M. Community-based research partnerships: challenges and opportunities. J Urban Health. (2005) 82(2 Suppl 2), ii3–12. 10.1093/jurban/jti034 15888635 PMC3456439

[9] Wallerstein N Duran B. Community-based participatory research contributions to intervention research: the intersection of science and practice to improve health equity. Am J Public Health. (2010) 100:S40–6. 10.2105/AJPH.2009.184036 20147663 PMC2837458

[10] Furco A. Advancing service-learning at research universities. N Direct Higher Educ. (2001) 2001:67. 10.1002/he.15.abs

[11] Nyden P. Academic incentives for faculty participation in community-based participatory research. J Gen Internal Med. (2003) 18:576–85. 10.1046/j.1525-1497.2003.20350.x 12848841 PMC1494894

[12] Marcus MT Taylor WC Hormann MD Walker T Carroll D. Linking service-learning with community-based participatory research: an interprofessional course for health professional students. Nurs Outlook. (2011) 59:47–54. 10.1016/j.outlook.2010.10.001 21256362

[13] Kronk R Weideman Y. Use of photovoice to integrate a community-engaged scholarship model of research into an undergraduate clinical nursing course. J Nurs Educ Thorofare. (2014) 53:S114–7. 10.3928/01484834-20140805-02 25081330

[14] Reising D Shea R Allen P Laux M Hensel D Watts P. Using service-learning to develop health promotion and research skills in nursing students. Int J Nurs Educ Scholarship. (2008) 5:29. 10.2202/1548-923X.1590 18673297

[15] Deale CS. Learning through engagement: Undergraduate students engaging in community-based participatory research (CBPR) in hospitality and tourism education. J Teach Travel Tourism. (2017) 17:55–61. 10.1080/15313220.2016.1270180

[16] Hammond J Hicks M Kalman R Miller J. PAR for the course: a congruent pedagogical approach for a PAR methods class. Michigan J Commun Serv Learn. (2005) 12:52–66.

[17] Martinez LS Perea FC Ursillo A Pirie A Ndulue UJ Oliveira C. Research as curriculum: engaging undergraduates and community residents in immigrant health research partnerships. Prog Commun Health Partnerships Res Educ Act. (2012) 6:491–8. 10.1353/cpr.2012.0059 23221295

[18] Jansen DA Jadack RA Ayoola AB Doornbos MM Dunn SL Moch SD. Embedding research in undergraduate learning opportunities. Western J Nurs Res. (2015) 37:1340–58. 10.1177/0193945915571136 25694176

[19] Undergraduate Public Health Learning Outcomes. Association of the Schools and Programs of Public Health (2011).

[20] Israel B Schulz A Parker E Becker A Allen A Guzman J. Critical issues in developing and following community-based participatory research principles. In: Minkler M, Wallerstein N, editors. Community-Based Participatory Research for Health. Jossey-Bass/Wiley (2003). p. 56–73.

[21] Zhang E Yigletu S Lieberman H Kosinski K Mukthineni R McLeod D. Perspectives on initiating community-based participatory research partnerships. J Commun Engag Scholarship. (2020) 12:55–63.

[22] Creswell JW Plano Clark VL. Designing and Conducting Mixed Methods Research. 2nd ed. Los Angeles, CA: Sage Publications (2011).

[23] Lichtenstein G Tombari M Thorme T Cutforth N. Development of a national survey to assess student learning outcomes of community-based research. J Higher Educ Outreach Engage. (2011) 15:7–34.

[24] StataCorp. Stata Statistical Software: Release 16. College Station, TX: StataCorp LLC (2019).

[25] Braun V Clarke V. Successful Qualitative Research: A Practical Guide for Beginners. London: SAGE (2013).

[26] QSR International Pty Ltd. NVivo (Version 12 Plus) (2018). Available online at: https://www.qsrinternational.com/nvivo-qualitative-data-analysis-software/home

[27] Kiviniemi MT Mackenzie SLC. Framing undergraduate public health education as liberal education: who are we training our students to be and how do we do that? Front Public Health. (2017) 5:9. 10.3389/fpubh.2017.00009 28239603 PMC5301016

[28] Auchincloss LC Laursen SL Branchaw JL Eagan K Graham M Hanauer DI. Assessment of course-based undergraduate research experiences: a meeting report. CBE Life Sciences Educ. (2014) 13:29–40. 10.1187/cbe.14-01-0004 24591501 PMC3940459

[29] Freeman S Eddy SL McDonough M Smith MK Okoroafor N Jordt H. Active learning increases student performance in science, engineering, and mathematics. Proc Natl Acad Sci USA. (2014) 111:8410–5. 10.1073/pnas.1319030111 24821756 PMC4060654

[30] Rodenbusch SE Hernandez PR Simmons SL Dolan EL. Early Engagement in course-based research increases graduation rates and completion of science, engineering, mathematics degrees. CBE Life Sci Educ. (2016) 15:ar20. 10.1187/cbe.16-03-0117 27252296 PMC4909342

[31] Yeatts KB. Active learning by design: an undergraduate introductory public health course. Front Public Health. (2014) 2:284. 10.3389/fpubh.2014.00284 25566526 PMC4275053

[32] Reinschmidt KM Maez P Iuliano JE Nigon BM. Using active learning strategies linked to CBPR principles in a semester-long class project to teach qualitative research methods in public health. Pedagogy Health Promot. (2019) 5:36–44. 10.1177/2373379918761976

[33] Trott CD Weinberg AE McMeeking LBS. Prefiguring sustainability through participatory action research experiences for undergraduates: reflections and recommendations for student development. Sustainability. (2018) 10:3332. 10.3390/su10093332

[34] Hunter AB Laursen SL Seymour E. Becoming a scientist: the role of undergraduate research in students' cognitive, personal, professional development. Sci Educ. (2007) 91:36–74. 10.1002/sce.20173

[35] Hathaway R Nagda B Gregerman S. The relationship of undergraduate research participation to graduate and professional education pursuit: an empirical study. J Coll Student Dev. (2002) 43:614–631.

[36] Carpi A Ronan DM Falconer HM Lents NH. Cultivating minority scientists: Undergraduate research increases self-efficacy and career ambitions for underrepresented students in STEM. J Res Sci Teach. (2017) 54:169–94. 10.1002/tea.21341

[37] Michaelsen LK Knight AB Fink LD editors. Team-Based Learning: A Transformative Use of Small Groups in College Teaching. 1st ed. Virginia: Stylus Publishing (2004).

[38] Burgess A Bleasel J Haq I Roberts C Garsia R Robertson T. Team-based learning (TBL) in the medical curriculum: Better than PBL? BMC Med Educ. (2017) 17:243. 10.1186/s12909-017-1068-z 29221459 PMC5723088

[39] Ofstad W Brunner LJ. Team-based learning in pharmacy education. Am J Pharmaceut Educ. (2013) 77:1–11. 10.5688/ajpe77470 PMC3663624 23716738

[40] Caldwell WB Reyes AG Rowe Z Weinert J Israel BA. Community partner perspectives on benefits, challenges, facilitating factors, and lessons learned from community-based participatory research partnerships in detroit. Prog Commun Health Partnerships Res Educ Act. (2015) 9:299–311. 10.1353/cpr.2015.0031 26412771

[41] Paul EL. Community-based research as scientific and civic pedagogy. Peer Rev. (2006) 8.

